# Using Household Expenditure Surveys for Comparable and Replicable Nutritional Analysis: Evidence from México

**DOI:** 10.3390/nu14173588

**Published:** 2022-08-31

**Authors:** Alan Hernández-Solano, Víctor Pérez-Hernández, Soraya Burrola-Méndez, Alejandra Aguirre, Jesús Gallegos, Graciela Teruel

**Affiliations:** 1 Instituto de Investigaciones para el Desarrollo con Equidad (EQUIDE), Universidad Iberoamericana, Prolongación Paseo de Reforma 880 Lomas de Santa Fe, Álvaro Obregón 01219, Ciudad de México, Mexico; 2 Departamento de Salud, Universidad Iberoamericana, Prolongación Paseo de Reforma 880 Lomas de Santa Fe, Álvaro Obregón 01219, Ciudad de México, Mexico; 3 Independent Researcher, Tlalnepantla 54060, Estado de México, Mexico; 4 Independent Researcher, Cuautitlán Izcalli 54740, Estado de México, Mexico

**Keywords:** micronutrients, household income expenditure surveys, calories, proteins, vitamins, iron, zinc, Mexico, food away from home

## Abstract

In this study, we explore how to use household expenditures and income surveys (HEIS) to provide replicable and comparable measures of nutrients availability at the population level. Our method formalizes the common practice in the literature and consists of three steps: identification of relevant food categories, pairing of food contents food groups in HEIS data, and calculation of the typical amount of nutrients by food group. We illustrate the usage of the method with Mexican data and provide a publicly available data set to readily convert food purchases into six nutrients: calories, proteins, vitamins A and C, iron, and zinc. We perform a descriptive analysis of the evolution of nutrients intake among Mexican households between 2008 and 2020, considering differences by income level. Our results reflect the effect of the COVID-19 pandemic on nutrient availability in Mexican households, mainly driven by a substantial reduction in the expenditure in food consumed away from home, although for most nutrients the trend was stable over most of the period.

## 1. Introduction

The analysis of nutrients availability and intake in the population is a key element of epidemiological surveillance and public policy design, particularly in the areas of food insecurity, obesity, malnutrition, and other problems associated with diet and food availability [1]. Evidence in developing countries indicates that inadequate nutrient intake is strongly associated with various health and non-health outcomes, such as developmental problems in childhood, obesity, anemia, and even labor productivity, all of which can influence aspects ranging from birth rates to economic growth [2,3]. Deficiencies in nutrient intake have long-term and even intergenerational effects since suffering from them in one generation affects the health and productivity of future generations [4].

One of the most important limitations in nutritional analysis at the population level is the lack of adequate information sources to assess the multiple social, economic, and cultural dimensions associated with food consumption or its availability. There are different approaches to assess household food availability and consumption, ranging from observed-weighed food records—considered the gold standard—to self-reporting by phone or email [1,5,6]. However, reduced availability of the most rigorous sources (such as 24-h recall surveys), as well as other methodological limitations of those already in existence (such as non-nationally representative samples), have created interest in using other sources on information for nutritional analysis, such as household income and expenditure surveys (HEIS) [1].

The literature on the advantages and shortcomings of household expenditure surveys to assess nutritional outcomes has shown that survey design is a crucial aspect on the adequacy of such instruments to measure nutritional indicators, but the wide availability of these surveys—particularly in low and middle-income countries—makes them a suitable source of data in the absence of other resources [6]. Indeed, in many countries these surveys are used to estimate nutritional intake, which in turn are used to define poverty lines [7] or measure food security [8]. However, the methodology to convert expenditures to nutritional indicators is far from standard, even within the same country [6,9]. A major limitation of these studies is the lack of a common approach to convert food purchases to nutrient availability, which creates a profusion of estimates from which it is difficult to draw clear public policy recommendations.

In the case of Mexico, the primary source of data for the analysis of diet and food intake is the National Health and Nutrition Survey (ENSANUT, by its acronym in Spanish) [10], which is the most comprehensive source of data on the state of health and nutrition of the Mexican population, as well as its determinants and performance of the health system. Despite the wealth of data offered by the ENSANUT, the information it provides on household food consumption and spending is limited, so it is not possible to analyze how food purchases and nutrients intake vary over other key factors behind nutritional results such as household income, food prices, or poverty status [11,12,13]. Faced with this limitation, various studies interested in the role of economic factors on nutritional outcomes in Mexico have opted for other sources of information, specifically, the National Survey of Income and Expenditures of Households (ENIGH, by its acronym in Spanish) [14,15,16,17] (see Appendix A for a brief description of this data set). Although several studies have used this approach [4,18,19,20], the method of conversion from food expenditures to nutrients availability differs widely between studies, even when using the same data source, creating confusion on how to provide comparable and replicable figures to address policy relevant questions.

In this article, we propose a methodology to measure in a comparable and replicable way the nutrient availability in household expenditure surveys. We illustrate the use of this methodology estimating the availability of nutrients in the ENIGH, the main source of information on household income and expenditure in Mexico [21]. Using our methodological approach, we introduce a new data set that can be used to estimate the availability of six nutrients (calories, proteins, vitamins A and C, iron, and zinc) with ENIGH data. The data set to make the conversions will be publicly available, so it can be used by interested researchers to readily transform ENIGH’s food expenditure data to nutrient availability. We perform a descriptive analysis of the evolution of nutrients intake among Mexican households between 2012 and 2020, considering differences by income level. Our results reflect the potential applications of our method and the data set of nutritional availability, by letting us follow the changes that the COVID-19 pandemic had on nutrient availability in Mexican households. Despite a substantial reduction in the expenditure in food consumed away from home, for most nutrients the trend has been stable over most of the period. These results highlight as well some of the limitations of household expenditures surveys to analyze nutritional indicators, particularly the bias associated to food consumed away from home, as well as the bias associated to large food acquisitions observed in previous studies [1,7,22,23,24].

## 2. Materials and Methods

Our methodological approach follows the common practice among the majority of studies that use HEIS to analyze nutritional outcomes [25]. However, unlike most previous studies, we propose a series of steps to systematize its implementation in other settings and facilitate comparability among studies. In addition, we implement this method to construct a conversion table of food purchases to nutrient availability that can be readily applied to analyze ENIGH data and will be publicly available to other researchers to facilitate the analysis of nutritional indicators with this data set.

### 2.1. Identification of Relevant Food Categories

Most HEIS provide a detailed account of food and beverages purchased by each household in a reference period, typically seven days, including the quantity acquired and the price paid. Since the original purpose of these instruments was to provide data for the construction and update of consumer price indices, items are usually classified into categories which may or may not have similar nutritional qualities. Thus, the main challenge to use HEIS data for nutritional analysis is to provide a convincing estimation of the amount of nutrients that each of these categories provide. The ENIGH, for example, contains 245 of such categories, although nine of these have no nutritional value (tobacco, in animal feed, food preparation, among others). Thus, a first step is to identify how food and beverage purchases are classified in the HEIS information, so that we have a clear idea of all the categories that need to be filled and ensure that we have data on the quantities bought in the reference period. Although other data may be relevant (such as prices, frequency of purchases, and so on), if we do not have access to the quantity available in each food category and the unit of measurement, we cannot implement this method.

In addition, many HEIS provide information on food consumed away from home, but usually we have only access to the total amount spent by the household, so that no details on the quantity or quality of the food consumed are available. This is a major limitation that will be explored later.

### 2.2. Pairing of Food Contents to Each Category in the HEIS Data

Once we know all relevant food categories, and we have data on the quantity purchased, we need to provide an estimation on the nutrients typically provided by the food purchased in each category. The HEIS food categories may combine types of foods with considerable variations in nutritional quality and presentations, so that producing a robust estimation of the nutritional value for each category requires a list of all commonly consumed foods and beverages, which contains its nutritional data. Typically, nutritional tables provide this information, although additional sources such as websites or scientific articles may be of use. Then, we sort all foods in the list into the HEIS categories and check that each category has an association. In case a certain category remains empty, we will need to repeat the previous process with additional sources of nutritional information. For all paired foods, data on the edible portion, as well as the contents of six nutrients (calories, proteins, vitamins A and C, iron, and zinc) were selected. In the case of vitamin A, we reported it as RAE (Retinol Activity Equivalents), which consider retinol in its main active form. It includes the conversion of beta-carotenes and other pro-vitamin A carotenoids into retinol.

There are several sources of nutritional data for foods and beverages, from those provided by official agencies like the United States Department of Agriculture (USDA) [26], to multiple internet sites such as www.dietas.net (accesed on 5 August 2022) or the FatSecret portal (which has several country-specific websites) [27,28]. However, due to cultural and geographical particularities, these sources are unlikely to contain information on all the food categories in a specific HEIS. Thus, we recommend looking for local or regional sources of nutritional information, especially those used more frequently among local nutritionists. Indeed, it is not uncommon to find variations in the amount of nutrients for the same foods, or that different researchers assign foods into distinct categories. To avoid such discrepancies, in our exercise, three researchers iteratively performed a match between the ENIGH expenditure categories, and the products contained in five nutritional tables (see Table 1).

In the first iteration, each researcher assigned the foods from the Table of Composition of Mexican Foods and Food Products [29] (SZ onwards) to each ENIGH category. Discrepancies were reviewed collectively, so that only when a unanimous decision was reached a certain food was assigned to the selected category. In cases where no consensus was achieved, the foods in question were left out of the analysis. In the second iteration, the previous process was repeated to look for foods that could be fitted in the empty categories using the table “Composición de Alimentos: Valor Nutritivo de los Alimentos de Mayor Consumo” (MZ onwards) [30]. Finally, the remaining categories were filled using the foods available in five other sources: “Sistema Mexicano de Alimentos Equivalentes” (SMAE) [31]; “Tabla de Composición de Alimentos de Centroamérica” (INCAP) [32]; the portal FoodCentral (USDA) [26], as well as online searches to find academic articles, recipes or other data to provide the nutritional contents as required.

### 2.3. Calculation of the Typical Amount of Nutrients by Food Category

The previous stage linked the foods and beverages contained in nutritional tables to the spending categories in the HEIS. However, we may find two additional problems: (a) missing information on nutrient content; and (b) how to deal with spending categories where more than one food item was paired. In the first case, food categories where all nutritional content was missing, went through the previous procedure again until data on all micronutrients were found.

In the case of multiple pairings, the information of multiple foods needs to be aggregated to obtain the typical nutritional content of the category. Although a straight option could have been to use the simple average, as certain food categories contained highly dissimilar elements, we recommend checking for the existence of outliers. In our exercise, we used Tukey’s fences criteria [33] to identify extreme values within each category and nutrient. Specifically, for a particular key and nutrient, we excluded nutrients contents that were outside the interval given by the formula:

[*Q_1* − 1.5 *IQR*, *Q_3* + 1.5 *IQR*],

where *Q_i* is the *i*-th quintile and *IQR* is the interquartile range. All nutritional contents were standardized to amounts of 100 g of edible portion beforehand to ensure comparability in portion size and nutrient content. In addition, food items with two or more micronutrient content identified as outliers were eliminated from the analysis.

The nutritional content of each spending category was defined as the average of each nutrient contribution of all food paired within the same category. The final data set with nutrient content per 100 g of edible portion can be consulted with the Supplementary Material of the article (Data Set S1: “Nutrients ENIGH-average”), as well as on Zenodo [34]. Additionally, since other measures of central tendency may be more adequate to estimate the contents of nutrients, we also publish the data set estimated by using the median of all types of foods in each group (Data Set S2: “Nutrients ENIGH-median” in the Zenodo repository [34]).

To illustrate the potential applications of this method, as well as that of the data set of nutrient availability, in the next section, we provide some descriptive results about the main trends in nutrient attainability in the Mexican population. The estimates are constructed using data about the edible portion and the amount of nutrients available in each spending category. We firstly obtain the product of the amount purchased in the reference period by the edible portion, and then multiplying the result by the nutrient content of the category. Then, we divide the result by seven to approximate the daily availability. Finally, we divide that amount by the number of equivalent adults in the household using the official Mexican scale of equivalences [35].

Our estimates consider only the nutrients from food purchased for consumption in the household, as no clear methodology exists to impute the quantity of micronutrients obtained from food consumed away from home (FAFH). However, it is important to keep in mind this bias. As seen in Table 2, more than 60 percent of the households spend a positive amount in FAFH, and this expenditure represents more than 20% of all food expenditure. These results highlight the key role of FAFH in household expenditure, as well as the urgency to dispose of better alternatives to estimate its contribution to nutrients consumption. However, the existing methodologies to approximate its nutritional contents have important limitations and have only been studied in the context of calorie intake [1].

The previous results indicate that excluding FAFH means that the data on the availability of nutrients in 2020 are not comparable to observations corresponding to other years, since they generate the erroneous impression of an increase in consumption. Thus, in this study, we report results only for food bought for consumption in the household.

All estimations consider the complex sampling design of the ENIGH sample, as implemented with Stata (version 16.0, College Station, TX, USA).

## 3. Results

**Figure 1:**
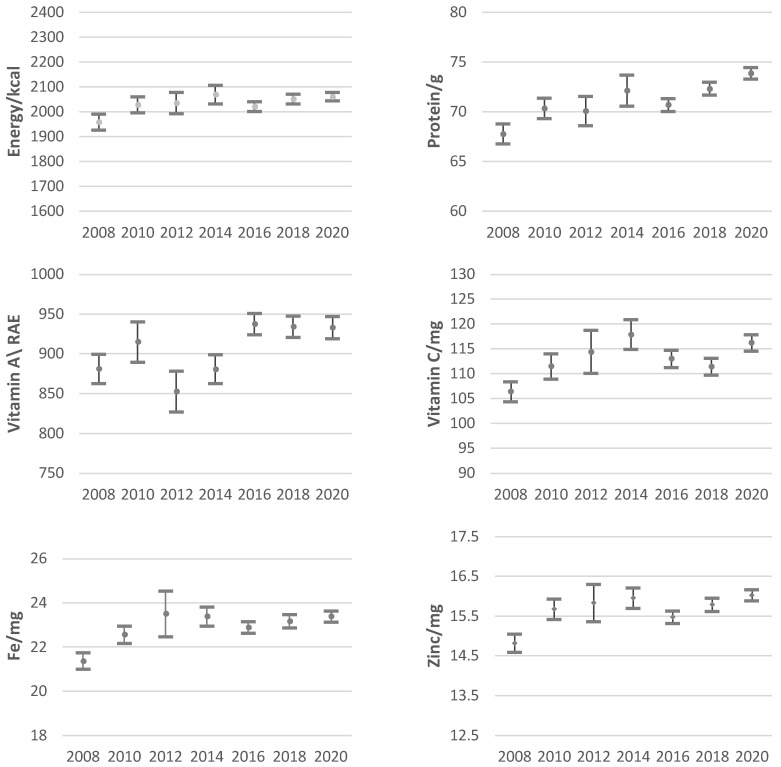
Daily nutrient availability per equivalent adult, 2008–2020. Confidence intervals at 95%. Source: Authors’ elaboration using data from the ENIGH [21] and the data set of nutrient availability elaborated by the authors.

Unlike previous studies [4,18], we find no clear trend in the evolution of daily per capita availability of nutrients over our period of interest (see Figure 1). Surprisingly, the consumption of all nutrients has remained stable during most of the period. In the case of the availability of energy, zinc, and vitamin A, it was similar during 2010–2020, while for vitamin C, protein, and iron, it was similar during the periods 2008–2018, 2010–2016, and 2010–2018. It is important to note that nutrient intake in 2020 was atypical due to the economic crisis stemming from the COVID-19 pandemic and other international factors.

Our estimations indicate that the acquisition of energy, iron, zinc, and vitamin A remained at approximately the same levels before and during the pandemic (in 2020), while the availability of proteins and vitamin C increased. Despite the exclusion of FAFH, the results on caloric intake are consistent with those of previous studies [4], which suggest that Mexican households were able to maintain their caloric intake in this period, even among those of lower resources.

Besides the temporal dimension, using ENIGH data let us explore interrelations with a wide array of socioeconomic indicators, particularly income, as shown in Table 3. Table 3 shows the availability of nutrients by income quintile in 2020, during the COVID-19 pandemic. In general, a positive relationship is observed between access to nutrients and income: in the case of calories, iron, and zinc, individuals in the lowest four quintiles had approximately the same level of nutrient availability, but all of them had lower availability than the population on the richest quintile. On the other hand, regarding proteins and vitamins, a strictly positive relationship was found: people in quintiles with higher income have, on average, a greater amount of proteins and vitamins. Furthermore, households in the richest quintile have 24, 46, and 83% more protein, and vitamins A and C, respectively, than those in the poorest.

In order to show the reliability of our results, estimates of nutrient availability are compared with calculations from previous articles (see Table 4), pointing out possible differences due to methodological discrepancies and discrepancies in information sources. Since several papers do not adjust their calculations for equivalence scales, our estimates of per capita nutrient availability without such adjustment are presented in Appendix B.

Regarding calories, it is found that the previous estimates based on the ENIGH are higher by 30–39% for the years 2008, 2010, 2012, and 2018. Except for 2012, the above can be justified because said works consider FAFH, while the present study does not. On the other hand, the results of [36] for 2012 should be considered with caution since, although they do not consider FAFH, their estimates are higher than those of other studies [4,18,37]. It was also found that the estimates on caloric availability of [19] for 2014 are 24% lower than those of this article, despite not considering FAFH too. Indeed, this effect arises because these authors could not find nutritional information for 40 ENIGH spending categories in the nutritional tables.

Our results are not directly comparable with those obtained from the ENSANUT since we approximate nutrient intake from food purchases. At the same time, the studies that use the ENSANUT employ the 24-h recall instrument. However, we compare both results to identify possible similarities between these estimates and found that, at the national level, the calculations on the availability of calories per capita in 2016 from the ENSANUT are higher than our estimates, which was expected given that the 24-h recall includes FAFH. For 2012, the comparisons yield mixed results: our estimates are similar for men aged 5–11 years and adolescent women, but lower for adolescent and adult men.

In relation to the estimates on the availability of proteins, previous estimates with the ENIGH for 2008 and 2010 are 27–45% higher than those found in this study since they consider the FAFH expenditure. In contrast, comparisons with estimates from the ENSANUT yield mixed results: our estimates are higher for all age groups except for adolescent males. On the other hand, estimates of the availability of vitamins and minerals were only identified using the ENSANUT. Our estimates for vitamin C were found to be very similar to those in the literature for 2012 and 2016, while those for vitamin A are higher. It was also observed that our estimates for iron and zinc are above those found from the ENSANUT.

## 4. Discussion

In this work we propose a methodology to convert data from HEIS to nutritional indicators that could be replicable and comparable among several settings. Although previous studies have used similar versions of this method, most of these studies base their analysis on a two-stage method, in which food intake is first estimated and then converted into nutrients using nutritional tables [4,18,20,41]. These studies emphasize the correct measurement of food intake, obviating that the use of different nutritional tables can generate considerable variations depending on multiple factors, such as the dates on which the food samples were analyzed and the bromatological tests, among others [41]. However, using nutritional tables relevant for the specific context of the study, complementing the data among several alternatives, and taking care on how multiple sources are correctly combined, have the potential to promote comparability among regions and studies.

The illustration with the case of Mexico provides an example of how using HEIS enables a wider array of analysis, by combining other socioeconomic variables, or even taking advantage of the sample design to make studies at the sub-national level. However, our analysis also reflects some of the usual problems in these studies: various analyses of nutritional indicators have been carried out based on the ENIGH [18,19,36,38,40], but there is little clarity about the criteria used for the construction of the input tables, as well as the potential biases that these differences in criteria create. For this reason, the table of nutrient contents introduced in this article provides a point of reference that gives certainty about the comparability of the results. This is a matter of great importance, since even the estimation of nutrients from the same table of contributions can generate substantial variability associated with the selection of food considered in each HEIS spending category [41].

The article uses the table of nutritional availability developed to estimate the intake of calories, proteins, vitamins (A and C), and minerals (iron and zinc) from 2008–2020, as well as throughout the income distribution in the year 2020. In general terms, the results indicate that per capita intake of all nutrients, adjusted for economies of scale, has remained stable throughout the period of analysis. Moreover, our estimations show that energy, iron, zinc, and vitamin A consumption remained stable during the pandemic, but that of protein and vitamin C increased considerably. Finally, a positive relationship was found between access to nutrients and income during 2020.

Finally, it should be mentioned the limitations of this methodological approach, as well as the results presented. One is that the estimated intake table is for raw foods, so when used to calculate nutrient intake, the loss of these at the time of cooking and preparation is not considered. This is particularly relevant in the case of vitamin C and Zinc, whose nutritional availabilities are known to be affected by cooking [38]. In addition, the calculation of vitamin A availability may be overestimated due to the large quantities of this nutrient in certain foods, hence the importance of considering other central tendency measures in future analysis. Another source of caution is that our calculations underestimate the availability of nutrients because they do not consider FAFH, so that future studies must develop appropriate methodologies to estimate the amount of nutrients from these sources. Finally, other well-known limitations arise from using information on food expenditures to approximate food consumption, such as the intra-household distribution of food, and the comparability of individuals from various age groups and ways of life, so that appropriate comparisons of nutritional availability may use alternative measures (such as adjusting nutritional content per 1000 kcal), among others [19].

## Figures and Tables

**Table 1:** Nutritional tables consulted and number of food pairings.

Nutritional Tables	Number of Foods Paired	Number of Categories Paired
SZ [29]	1023	185
MZ [30]	135	28
SMAE [31]	11	8
INCAP [32]	9	6
USDA [26]	5	5
Other sources *	7	5
*All pairings*	*1190*	*237*

*Notes*: The abbreviations correspond to each of the nutritional tables consulted: SZ for the Table of Composition of Mexican Foods and Food Products [29]; MZ for the “Composición de Alimentos: Valor Nutritivo de los Alimentos de Mayor Consumo” [30]; SMAE for the “Sistema Mexicano de Alimentos Equivalentes” [31]; INCAP for the “Tabla de Composición de Alimentos de Centroamérica” [32]; and, USDA for the portal FoodCentral [26]. The sum of the paired keys does not coincide with the number of categories analyzed (236), since in the case of categories A201 (“Barbacoa y birria”) and A223 (“Cognac y brandy”), we combined information from two nutritional tables. In categories corresponding to food packages—A242 and A212—the basic care packaged selected was the one available at SAM’s store web page. * For a limited number of categories, especially those of prepared meals, we did online searches to find academic articles, recipes, or other data to provide the nutritional contents as required. Source: Authors’ elaboration.

**Table 2:** Evolution of the proportion of households and average daily expenditure on food, food for consumption in the household, and food consumed away from home, Mexico 2014–2020.

Year	Households with Consumption of Food Away from Home		Daily per Capita Expenditure in Food for Consumption in the Household ^1,2^		Daily per Capita Expenditure in Food ^1,2^		Proportion of Total Food Expenditure Spent on Food Away from Home	
	%	IC 95%	MX$	IC 95%	MX$	IC 95%	%	IC 95%
2014	62.28	(61.03, 63.54)	33.12	(32.58, 33.65)	49.52	(48.54, 50.50)	22.73	(22.13, 23.33)
2016	68.65	(68.02, 69.27)	34.25	(33.92, 34.59)	54.35	(53.73, 54.97)	26.41	(26.05, 26.76)
2018	68.07	(67.45, 68.69)	35.17	(34.83, 35.51)	55.79	(55.12, 56.46)	26.48	(26.11, 26.85)
2020	53.78	(53.18, 54.38)	38.32	(37.98, 38.67)	52.99	(52.47, 53.51)	19.62	(19.32, 19.92)

Notes: ^1^ Constant prices of 2018. ^2^ Adjusted for economies of scale. Source: Authors’ elaboration using ENIGH data.

**Table 3:** Average daily nutrient availability per capita, adjusted by equivalence scales, by income quintiles (Mexico, 2020).

Quintile	Energy/kcal	Protein/g	VitA/RAE	VitC/mg	Fe/mg	Zinc/mg
I	2050.97	67.61	755.88	84.51	23.44	15.66
	(2011.92, 2090.03)	(66.51, 68.71)	(740.20, 771.55)	(82.64, 86.38)	(22.99, 23.88)	(15.40, 15.93)
II	2001.59	69.74	875.54	99.56	22.84	15.54
	(1972.93, 2030.25)	(68.84, 70.63)	(856.38, 894.70)	(97.42, 101.71)	(22.43, 23.24)	(15.31, 15.76)
III	2000.89	72.02	928.20	114.63	22.81	15.69
	(1973.67, 2028.11)	(71.12, 72.93)	(906.63, 949.77)	(111.77, 117.49)	(22.36, 23.26)	(15.46, 15.93)
IV	2049.98	75.93	1003.78	128.04	22.83	15.94
	(2019.04, 2080.91)	(74.86, 76.99)	(975.28, 1032.27)	(124.81, 131.28)	(22.41, 23.25)	(15.70, 16.19)
V	2202.20	84.14	1105.25	154.86	25.03	17.30
	(2157.96, 2246.43)	(82.49, 85.80)	(1057.74, 1152.76)	(149.90, 159.81)	(24.34, 25.73)	(16.92, 17.67)

Notes: Confidence intervals 95% in parentheses. Source: Authors’ elaboration using ENIGH 2020 data and CONEVAL equivalence scales [35].

**Table 4:** Previous estimates of nutritional availability and intake, México 2008–2018.

Nutrient	Source	Data	FAFH	Year	Population	Estimate
Energy/kcal	[18]	ENIGH	Yes	2008	Rural	2454
					Urban	2512
				2010	Rural	2485
					Urban	2444
	[3]	ENIGH ^A^	Yes	2008	National	2699
				2010	National	2683
	[36]	ENIGH	No	2012	National	2771
	[37]	ENSANUT	Yes	2012	Preschool-aged children (1–4 years)	1380
					School-aged boys (5–11 years)	1910
					School-aged girls (5–11 years)	1770
					Adolescent males (12–19 years)	2360
					Adolescent females (12–19 years)	1900
					Adult men (>=20 years)	2030
					Adult women (>=20 years)	1778
	[19]	ENIGH ^A^	No	2014	National	1581
	[38]	ENSANUT	Yes	2016	National	1903
	[39]	ENIGH ^A^	Yes	2018	National	2845
Protein/g	[18]	ENIGH	Yes	2008	Rural	84
					Urban	93
				2010	Rural	82
					Urban	94
	[37]	ENSANUT	Yes	2012	Preschool-aged children (1–4 years)	48
					School-aged boys (5–11 years)	63
					School-aged girls (5–11 years)	60
					Adolescent males (12–19 years)	78
					Adolescent females (12–19 years)	61
					Adult men (>=20 years)	75
					Adult women (>=20 years)	62
VitA/RAE	[40]	ENSANUT	Yes	2012	Preschool-aged children (1–4 years)	563
					School-aged boys (5–11 years)	597
					School-aged girls (5–11 years)	578
					Adolescent males (12–19 years)	584
					Adolescent females (12–19 years)	532
					Adult men (>=20 years)	547
					Adult women (>=20 years)	536
	[38]	ENSANUT	Yes	2016	National	553.1
VitC/mgg	[40]	ENSANUT	Yes	2012	Preschool-aged children (1–4 years)	98.3
					School-aged boys (5–11 years)	107
					School-aged girls (5–11 years)	112
					Adolescent males (12–19 years)	109
					Adolescent females (12–19 years)	111
					Adult men (>=20 years)	107
					Adult women (>=20 years)	101
	[38]	ENSANUT	Yes	2016	National	102.6
Fe/mg	[20]	ENSANUT	Yes	2012	Preschool-aged children (1–4 years)	10.3
					School-aged boys (5–11 years)	13.2
					School-aged girls (5–11 years)	13.2
					Adolescent males (12–19 years)	15.4
					Adolescent females (12–19 years)	15.4
					Adult men (>=20 years)	13.6
					Adult women (>=20 years)	11.5
Zinc/mg	[20]	ENSANUT	Yes	2012	Preschool-aged children (1–4 years)	8.00
					School-aged boys (5–11 years)	10.1
					School-aged girls (5–11 years)	9.47
					Adolescent males (12–19 years)	12.5
					Adolescent females (12–19 years)	9.28
					Adult men (>=20 years)	11.4
					Adult women (>=20 years)	9.27
	[38]	ENSANUT	Yes	2016	National	9

Note: ^A^ It considers the equivalence scales. Source: Authors’ elaboration.

## Data Availability

Data set of ENIGH conversion to nutrients available at a public repository https://doi.org/10.5281/zenodo.6984024 (accessed on 30 august 2022).

## Supplementary Materials

The following supporting information can be downloaded at: https://www.mdpi.com/article/10.3390/nu14173588/s1, Data Set S1: “Nutrients ENIGH-average”, Data Set S2: “Nutrients ENIGH-median”.

## Appendix A

The ENIGH is a biannual multipurpose survey produced by the Mexican National Institute of Statistics (INEGI) since 1992. Although the initial objective of the ENIGH was to provide inputs for the calculation and updating of the National Price Index for the Consumer, currently it is the main source of information for the official figures on multidimensional poverty and inequality, as well as household characteristics and other socioeconomic indicators [21]. The high level of detail with which the ENIGH captures household food spending has been used by previous studies to carry out studies on nutritional and economic issues. It has been used to generate statistical evidence on the effect of income and schooling on energy and micronutrient consumption. Moreover, it has made it possible to analyze the relationship between objective and subjective food safety measures, quantifying the former from the caloric content and/or the diversity of the diet, and the latter through the Mexican Scale of Food Security [19,36].

## Appendix B

**Table A1:** Daily nutrient availability per capita in Mexico, 2008–2020.

Year	Energy/kcal	Protein/g	VitA/RAE	VitC/mg	Fe/mg	Zinc/mg
2008	1888.1	65.3	847.4	102.2	20.6	14.3
	(1855.2, 1921.0)	(64.3, 66.4)	(829.3, 865.4)	(100.2, 104.2)	(20.2, 21.1)	(14.0, 14.5)
2010	1860.0	64.6	842.1	102.9	20.7	14.4
	(1829.7, 1890.4)	(63.6, 65.5)	(818.2, 866.0)	(100.5, 105.2)	(20.3, 21.1)	(14.1, 14.6)
2012	1872.1	64.5	787.3	106.1	21.7	14.6
	(1832.3, 1911.8)	(63.1, 65.9)	(763.2, 811.4)	(102.0, 110.2)	(20.7, 22.6)	(14.1, 15.0)
2014	1907.0	66.5	813.7	109.5	21.5	14.7
	(1870.8, 1943.1)	(65.0, 68.1)	(796.6, 830.8)	(106.6, 112.4)	(21.1, 22.0)	(14.5, 14.9)
2016	1867.9	65.4	869.4	105.2	21.1	14.3
	(1849.1, 1886.8)	(64.8, 66.0)	(856.6, 882.2)	(103.5, 106.8)	(20.9, 21.4)	(14.1, 14.4)
2018	1904.9	67.2	869.5	104.1	21.5	14.6
	(1886.3, 1923.6)	(66.6, 67.8)	(856.5, 882.4)	(102.5, 105.8)	(21.2, 21.8)	(14.5, 14.8)
2020	1927.1	69.1	875.0	109.3	21.9	15.0
	(1910.6, 1943.7)	(68.6, 69.7)	(861.4, 888.7)	(107.7, 110.9)	(21.6, 22.1)	(14.9, 15.1)

Notes: Confidence intervals 95% in parentheses. Source: Authors’ elaboration using ENIGH 2020 data and CONEVAL equivalence scales [32].
