# Supplementary material for: Using Household Expenditure Surveys for Comparable and Replicable Nutritional Analysis: Evidence from México
Source: Nutrients. 2022 Aug 31;14(17):3588. doi: 10.3390/nu14173588 (PMC9460767; doi:10.3390/nu14173588)
Supplement: Supplementary file 1 [file nutrients-14-03588-s001.zip › Descripci¿«n BD.pdf]

Investigación con Impacto Social

NUTRITIONAL TABLE TO ESTIMATE THE AVAILABILITY OF NUTRIENTS IN HOUSEHOLDS FROM THE MEXICAN NATIONAL SURVEY OF HOUSEHOLD INCOME AND EXPENDITURES (ENIGH) 2008-2020

DATABASE DESCRIPTION

t. 5950.4339
Prolongación Paseo de la Reforma 880
Lomas de Santa Fe, México, C.P. 01219

CONTENIDOS DE LA BASE DE DATOS

The data set "Nutrients ENIGH-average" contains information on edible portions and the content of six nutrients (calories, proteins, vitamins A and C, iron, and zinc) in 100 grams of the edible portion of ENIGH's expenditure categories.

That data set was estimated using the average as a measure of central tendency (see methodology section in the file "READ ME.txt"). Additionally, the data set "Nutrients ENIGH-median" was calculated using the median.

Both data sets have the same structure and variables described in the following table:

Variable	Description
ENIGH's Code	Food code according to ENIGH catalog of expenses (see https://www.inegi.org.mx/app/biblioteca/ficha.html?upc=889463901242)
ENIGH's Food Categories	Food code description
Edible Portion (%)	Percent of edible portion
Energy/kcal	Content of calories (kcal) in 100 grams of the edible portion
Protein/g	Content of proteins (g) in 100 grams of the edible portion
Fe/mg	Content of iron (mg) in 100 grams of the edible portion
Zinc/mg	Content of zinc (mg) in 100 grams of the edible portion
VitA/µg RAE	Content of vitamin A (µg RAE) in 100 grams of the edible portion
VitC/mg	Content of vitamin C (mg) in 100 grams of the edible portion
