# Supplementary material for: Using Household Expenditure Surveys for Comparable and Replicable Nutritional Analysis: Evidence from México
Source: Nutrients. 2022 Aug 31;14(17):3588. doi: 10.3390/nu14173588 (PMC9460767; doi:10.3390/nu14173588)
Supplement: Supplementary file 1 [file nutrients-14-03588-s001.zip › READ ME.pdf]

Title: Nutritional table to estimate the availability of nutrients in households from the Mexican National Survey of Household Income and Expenditures (ENIGH) 2008-2020

Título: Tabla de aportes para estimar la disponibilidad de nutrientes en hogares de la Encuesta Nacional de Ingresos y Gastos de los Hogares (ENIGH) 2008-2020

How to cite
Hernandez-Solano, Alan; Perez-Hernandez, Victor; Burrola-Méndez, Soraya; Aguirre, Alejandra; Gallegos, Jesús; Teruel-Belismelis, Graciela. (2022) Tabla de aportes para estimar la disponibilidad de nutrientes en hogares de la Encuesta Nacional de Ingresos y Gastos de los Hogares (ENIGH) 2008-2020. [Data set]. EQUIDE-UIA.

Regional Baskets for Poverty Measurement Project
The database makes it possible to transform the ENIGH's information on the quantities (kilograms or liters) of food purchased by households into six nutrients: calories, proteins, vitamins A and C, iron, and zinc.

Language:
The dataset is in English.

Dataset format:
The dataset is presented in ".csv" format, which eases the preservation in the repository and amplifies the number of statistical packages that can read it.

Methodology:
The nutrient content (calories, proteins, vitamins A and C, iron, and zinc) and the edible portion of ENIGH's expenditure items were obtained iteratively from different sources. In the first iteration, three researchers assigned the food from the Content Table of Mexican Food and Food Products [1] to each ENIGH category.
Discrepancies were collectively reviewed so that only when a unanimous decision was reached a certain food was assigned to the selected category. In cases without consensus, the food in question was left out of the analysis.
In the second iteration, the previous process was repeated to look for food that could be fitted in the empty categories using the table "Composición de Alimentos: Valor Nutritivo de los Alimentos de Mayor Consumo" [2].
Finally, the remaining categories were filled using the food available in five other sources: "Sistema Mexicano de Alimentos Equivalentes" (SMAE) [3]; "Tabla de Composición de Alimentos de Centroamérica" (INCAP) [4]; the portal FoodCentral (USDA) [5], as well as online search to find academic articles, recipes or other data to provide the nutritional contents as required.

In the case of multiple pairings, the information of multiple foods should be aggregated to obtain the typical nutritional content of the category. Although a straight option could have used the simple average, as certain food categories contained highly dissimilar elements, we recommend checking

for the existence of outliers. Our exercise used Tukey's fences criteria [6] to identify extreme values within each category and nutrient.
For a particular key and nutrient, we excluded nutrient contents that were outside the interval given by the formula:
[Q_1-1.5 IQR, Q_3+1.5 IQR], where Q_i is the i-th quintile and IQR is the interquartile range.

All nutritional contents were standardized to amounts of 100 grams of edible portion beforehand to ensure comparability in portion and nutrient content. In addition, food items with two or more micronutrient contents identified as outliers were eliminated from the analysis. Finally, the nutritional content of each spending category was defined as the average of each nutrient contribution of all food paired within the same category.
Also, since other measures of central tendency different from the average could be considered, we generated the data set using the median. For further details on the methodology, see the published article.

References
1.- J. C. Morales de León, H. Bourges-Rodríguez, y M. E. Camacho-Parra, Tables of composition of foods and food products (condensed version 2015). Ciudad de México: Instituto Nacional de Ciencias Médicas y Nutrición Salvador Zubirán, 2016. Accedido: 29 de junio de 2022. [En línea]. Disponible en: https://www.incmnsz.mx/2019/TABLAS_ALIMENTOS.pdf
2.- M. Muñoz de Chávez y J. A. Ledesma Solano, Composición de alimentos: valor nutritivo de los alimentos de mayor consumo, 2.a ed. México, Ciudad de México: McGraw-Hill, 2010.
3.- A. B. Pérez Lizaur, B. Palacios González, A. L. Castro Becerra, y I. Flores Galicia, Sistema mexicano de alimentos equivalentes. Cuadernos De Nutricion (Fomento de Nutricion y Salud), 2014.
4.- Instituto de Nutrición de Centro América y Panamá, Tabla de composición de alimentos de Centroamérica, 2.a ed. Guatemala: INCAP/OPS, 2007. Accedido: 28 de junio de 2022. [En línea]. Disponible en: http://www.incap.int/mesocaribefoods/dmdocuments/TablaCAlimentos.pdf
5.- USDA, «FoodData Central», Food Data Central, 2019. https://fdc.nal.usda.gov/ (accedido 29 de junio de 2022).
6.- J. W. Tukey, Exploratory Data Analysis. Reading, MA: Addison-Wesley, 1977.

Results
The data set "Nutrients ENIGH-average" contains information on edible portions as well as the content of six nutrients (calories, proteins, vitamins A and C, iron, and zinc)
in 100 grams of the edible portion of ENIGH’s expenditure categories.
That data set was estimated using the average as a measure of central tendency (see methodology section).
Additionally, the data “Nutrients ENIGH-median” was calculated using the median.

#Contact
If researchers require additional information, please contact Victor Perez (victor.hernandez@ibero.mx) or Alan Hernandez (alan.hernandez@ibero.mx)
